# Supplementary material for: The trans-zeatin-type side-chain modification of cytokinins controls rice growth
Source: Plant Physiol. 2023 Mar 30;192(3):2457–74. doi: 10.1093/plphys/kiad197 (PMC10315312; doi:10.1093/plphys/kiad197)
Supplement: kiad197_Supplementary_Data [file kiad197_supplementary_data.pdf]

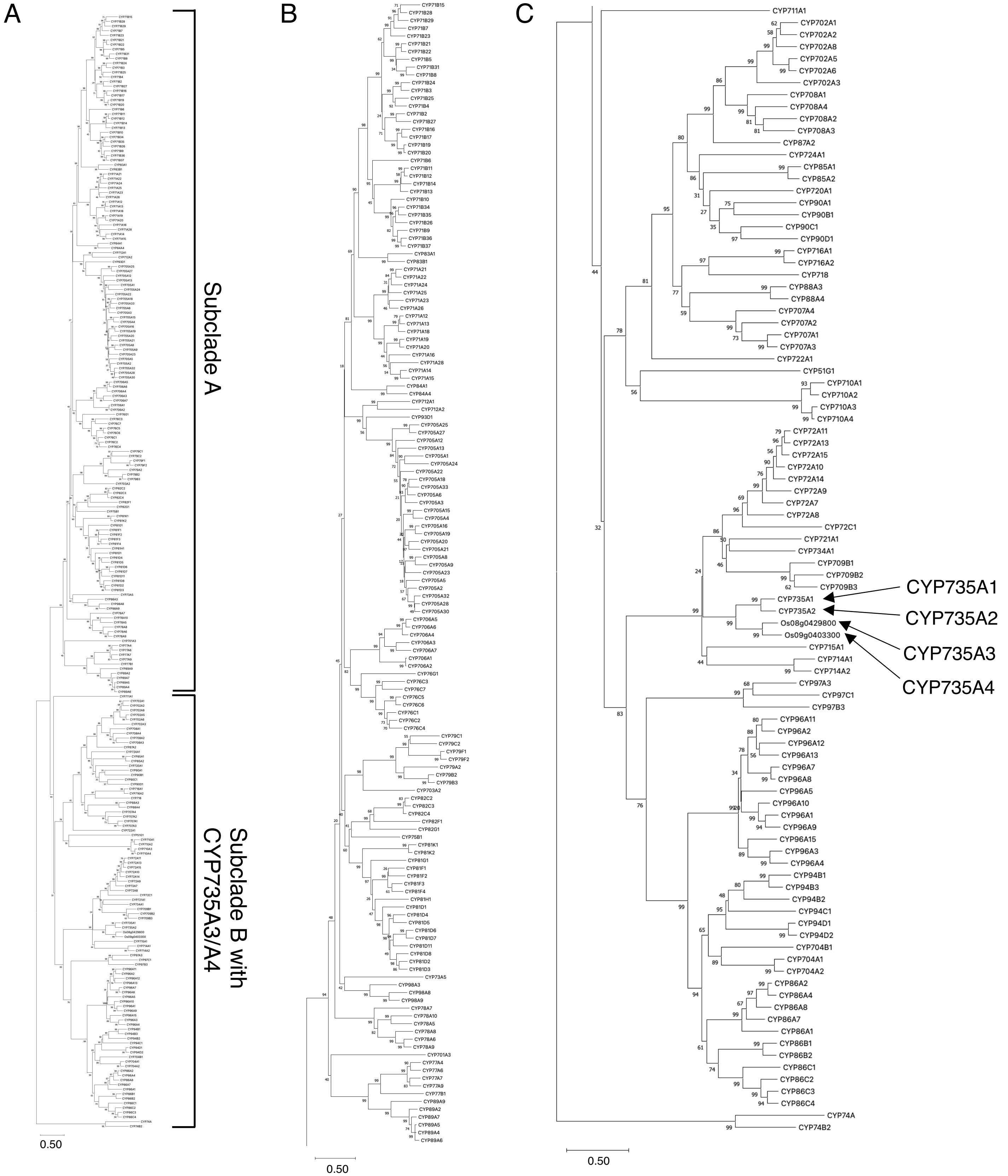

Supplemental Figure S1. Phylogenetic tree of Arabidopsis cytochrome P450s, CYP735A3 and CYP735A4

(A) A phylogenetic tree of 293 Arabidopsis P450s, CYP735A3 (LOC_Os08g33300/Os08g0429800) and CYP735A4 (LOC_Os09g23820/Os09g0403300). (B) Enlargement of subclade A. (C) Enlargement of subclade B with CYP735A3 and CYP735A4. Full-length amino acid sequences were obtained from Plant P450 database (https://erda.dk/public/vgrid/PlantP450/index.html). A phylogenetic tree was inferred by the Neighbor-Joining method using MEGAX (https://www.megasoftware.net/). The tree is drawn to scale, with branch lengths in the same units as those of the evolutionary distances used to infer the phylogenetic tree. The evolutionary distances were computed using the JTT matrix-based method and are in the units of the number of amino acid substitutions per site. All positions containing gaps and missing data were eliminated. The values at the nodes indicate the bootstrap values (using 500 replications). The scale bars represent the genetic distance measured in the number of substitutions per site.

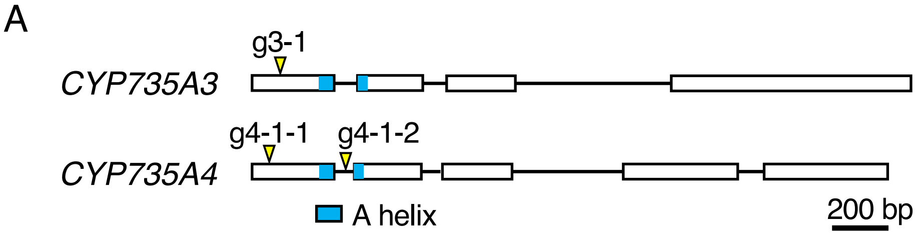

B

```
                  g3-1
CYP735A3:  79 ACCGTCTCATGCTACTACCTCA-CGCCGGCGAGG 111
a3-1    :     ACCGTCTCATGCTACTACCTCACCGCCGGCGAGG
```

C

```
                      g4-1-1
CYP735A4: 46   CTCGTGGCGCTGCTTCTGAGGGCGGCGTGGGTGACCCTGT 85
a4-1    :      CTCGTGGCGCTGCTTCTGA---------------------

CYP735A4: 86   CCTGCTACTGGCTGACGCCAATGAGGATCCGCCGCGCCAT 125
a4-1    :      ----------------------------------------

CYP735A4: 126  GGCGGCGCAGGGCGTGCGCGGCCCGCCGCCGCGCCCGCTC 165
a4-1    :      ----------------------------------------

CYP735A4: 166  GTCGGCAACCTCCGGGAGGTGTCGGCGCTCGTGGCGAGGG 205
a4-1    :      ----------------------------------------

CYP735A4: 206  CCACCGCCGACGACATGCCGTCCCTCAGCCACGACATCGT 245
a4-1    :      ----------------------------------------

CYP735A4: 246  CGGCCGCCTCATGCCCCACTACGTGCTCTGGTCCGGGACA 285
a4-1    :      ----------------------------------------

CYP735A4: 286  TACGGTGAGCACGCCACGCGCGCGACACGCCGCCGCCGGC 325
a4-1    :      ----------------------------------------
                    g4-1-2
CYP735A4: 326  GATTGTTCGTTCGTTCGTTGACTCTTTTTGGTTTGCAGGC 365
a4-1    :      --TTGTTCGTTCGTTCGTTGACTCTTTTTGGTTTGCAGGC
```

Supplemental Figure S2. The *cyp735a3-1 cyp735a4-1* mutant generated by the CRISPR/Cas9 system
(A) Schematic representation of CRISPR target sites to generate *cyp735a3-1* (*a3-1*) and *cyp735a4-1* (*a4-1*) alleles. Boxes represent exons; horizontal bars, introns; triangles, CRISPR target sites. The blue box represents the "A helix" . The bar indicates a 200 bp scale. (B) Partial sequences of wild-type *CYP735A3* and *cyp735a3-1* (*a3-1*) mutant. The sequence corresponding to a guide RNA used to generate the *a3-1* mutation (g3-1) is highlighted in yellow. (C) Partial sequences of wild-type *CYP735A4* and *cyp735a4-1* (*a4-1*) mutant. The sequence corresponding to guide RNAs used to generate the *a4-1* mutation (g4-1-1 and g4-1-2) is highlighted in yellow. Numbers in (B) and (C) represent positions in a genome sequence when the first nucleotide of the putative start codon is counted as 1. The red letter and red dash indicate an inserted and deleted sequence, respectively.

A

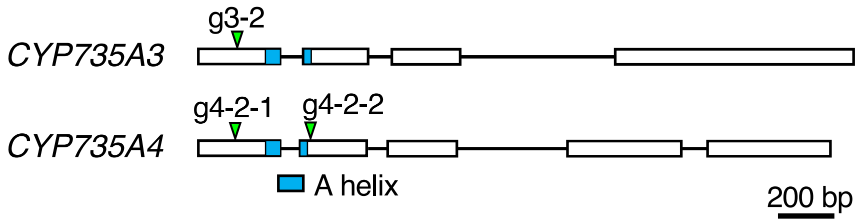

B

```
                                   g3-2
CYP735A3: 110 GGATCCGGCGGGTCCTGGCGAG-CCAGGGCGTGC 142
a3-2    :     GGATCCGGCGGGTCCTGGCGAGACCAGGGCGTGC
```

C

```
                        g4-2-1
CYP735A4: 86  CCTGCTACTGGCTGACGCCAATGAGGATCCGCCGCGCCAT 125
a4-2    :     CTCGTGGCGCTGCTTCTGA---------------------

CYP735A4: 126 GGCGGCGCAGGGCGTGCGCGGCCCGCCGCCGCGCCCGCTC 165
a4-2    :     ----------------------------------------

CYP735A4: 166 GTCGGCAACCTCCGGGAGGTGTCGGCGCTCGTGGCGAGGG 205
a4-2    :     ----------------------------------------

CYP735A4: 206 CCACCGCCGACGACATGCCGTCCCTCAGCCACGACATCGT 245
a4-2    :     ----------------------------------------

CYP735A4: 246 CGGCCGCCTCATGCCCCACTACGTGCTCTGGTCCGGGACA 285
a4-2    :     ----------------------------------------

CYP735A4: 286 TACGGTGAGCACGCCACGCGCGCGACACGCCGCCGCCGGC 325
a4-2    :     ----------------------------------------

CYP735A4: 326 GATTGTTCGTTCGTTCGTTGACTCTTTTTGGTTTGCAGGC 365
a4-2    :     ----------------------------------------

CYP735A4: 366 AAGCTGTTCGTGTACCTGTACGGGAGCGAGCCGAGGCTGT 405
a4-2    :     ------------------------------------CTGT
                  g4-2-2
CYP735A4: 406 GCCTGACTGACACCGCGCTGATCAAGGAGTTCTTGTCGTC 445
a4-2    :     GCCTGACTGACACCGCGCTCTGTGCCTGACTGACACCGCG
```

Supplemental Figure S3. The *cyp735a3-2 cyp735a4-2* mutant generated by CRISPR/Cas9 system
(A) Schematic representation of CRISPR target sites to generate *cyp735a3-2* (*a3-2*) and *cyp735a4-2* (*a4-2*) alleles. Boxes represent exons; horizontal bars, introns; triangles, CRISPR target sites. The blue box represents the “A helix”. The bar indicates a 200 bp scale. (B) Partial sequences of wild-type *CYP735A3* and *cyp735a3-2* (*a3-2*) mutant. The sequence corresponding to a guide RNA used to generate the *a3-2* mutation (g3-2) is highlighted in yellow. (C) Partial sequences of wild-type *CYP735A4* and *cyp735a4-2* (*a4-2*) mutant. The sequence corresponding to guide RNAs used to generate the *a4-2* mutation (g4-2-1 and g4-2-2) is highlighted in green. Numbers in (B) and (C) represent positions in a genome sequence when the first nucleotide of the putative start codon is counted as 1. The red letter and red dash indicate an inserted and deleted sequence, respectively.

```
                                                          g3-1     g3-2
                                                            ▼        ▼
CYP735A3     MAAAVLVAIALPVSLALLLVAKAVWVTVSCYYLTPARIRRVLASQG--------------    46
a3-1         MAAAVLVAIALPVSLALLLVAKAVWVTVSCYYLTAGEDPAGPGEPGRARPAAAAARRQPP    60
a3-2         MAAAVLVAIALPVSLALLLVAKAVWVTVSCYYLTPARIRRVLARPGRARPAAAAARRQPP    60
             ********************************** ..     .  *

CYP735A3     --------------------VRGPPP---RPLVGNLRDVSALVAESTAADMASL-SH---    79
a3-1         RRVGARRRVHRRRHGLPQPRHRRPPPPPLRPLVQHVREAVRVLVRERAAGVRDGGRHGAG    120
a3-2         RRVGARRRVHRRRHGLPQPRHRRPPPPPLRPLVQHVREAVRVLVRERAAGVRDGGRHGAG    120
                                   * ***   **** ::*:.  ::... **.: .   *

CYP735A3     -DIVARLLPHYVLWSNTYGRRFVYWYGSEPRVCVTEAGMVRELLSSRHAHVTGKSWLQRQ    138
a3-1         APVVAARARHRQVVAAAAG-----------------------------RQALHRPWPPHG    151
a3-2         APVVAARARHRQVVAAAAG-----------------------------RQALHRPWPPHG    151
               :**    *  : : : *                               :.  : *  :

CYP735A3     GAKHFIGRGLLMANGATWSHQRHVVAPAFMAD-RLKG--------RVGHMVECTRQTVRA    189
a3-1         -------------------QRRHLVAPAPRRRAGVHGRPAQGEGGAHGGVHEADGAGA--    190
a3-2         -------------------QRRHLVAPAPRRRAGVHGRPAQGEGGAHGGVHEADGAGA--    190
                                ::**:****      ::*           * : *.    .

CYP735A3     LRDAVARSGNEVEIGAHMARLAGDVIARTEFDTSYETGKRIFLLIEE-LQRLTARSSRYL    248
a3-1         -----EG------CGGEVRERGGDRRAHGEARRRRDRAHRVRHELRDRQEDLPPHR--GA    237
a3-2         -----EG------CGGEVRERGGDRRAHGEARRRRDRAHRVRHELRDRQEDLPPHR--GA    237
                           *..: . .**  *: *     : .:*:   :.:  : *  :

CYP735A3     WVPGSQYFPSKYRREIKRLNGELERLLKESIDRSRE-------IADEG---RTPSASPCG    298
a3-1         PAPHRPLQPLP--------LGPRQPVFSEQVQERDKAAERRAGAAAQGVHRPEPGDRRRG    289
a3-2         PAPHRPLQPLP--------LGPRQPVFSEQVQERDKAAERRAGAAAQGVHRPEPGDRRRG    289
              .*     *            *  : ::.*.::.  :        * :*     *.    *

CYP735A3     RGLLGMLLAEMEKKEAGGNGGGE---VGYDAQMMIDECKTFFFAGHETSALLLTWAIMLL    355
a3-1         PDAVGVAVRPWPPRHAAGRDGEEGGRRQWRRRGRVRRPD----DDRRVQDLLLRRPR-DV    344
a3-2         PDAVGVAVRPWPPRHAAGRDGEEGGRRQWRRRGRVRRPD----DDRRVQDLLLRRPR-DV    344
              . :*: :     :.*.*..* *     :  :  :  . .     .:... ***       :

CYP735A3     ATHPAWQDKARAEVAAVCGGGAPSPDSLPKLAVLQMVINETLRLYPPAT-LLPRMAFEDI    414
a3-1         GAAPHLGHHAARHAPGVAGQGARRGRR--RLRRR-----RAVAGQPPEARRAPDGDQ---    394
a3-2         GAAPHLGHHAARHAPGVAGQGARRGRR--RLRRR-----RAVAGQPPEARRAPDGDQ---    394
             .: *   .:*  .. .*.* **       :*         .::    ** :    *

CYP735A3     ELGGGALRVPSGASVWIPVLAIH------HDEGAWGRD-----AHEFRPDRFAPGRPRPP    463
a3-1         ---RDAAAVPAGDAAAADGVRGHRARRGRAPGAEWRVGVDPGARHPPRRGRVGPRRARVQ    451
a3-2         ---RDAAAVPAGDAAAADGVRGHRARRGRAPGAEWRVGVDPGARHPPRRGRVGPRRARVQ    451
                 .*  **:* :.    :  *          . *  .       *  * .*..* * *

CYP735A3     AGAFLPFAAGPRNCVGQAYAMVEAKVALAMLLSSFRF-AISDEYRHAPVNVLTLRPRHGV    522
a3-1         AGQVR---AGTAAAAGGGVPAVRRRAAQ-LRRAGVRHGGGQGRARHAPLQL-PLRHLRRV    506
a3-2         AGQVR---AGTAAAAGGGVPAVRRRAAQ-LRRAGVRHGGGQGRARHAPLQL-PLRHLRRV    506
             ** .    **   ..* .   *. :.*  :  :..*. . ... ****:::  **  : *

CYP735A3     PVRL-----LP------LPPPRP-------   534
a3-1         PARAGERAHAPATPRRARPPPAAAAAAAPIX   536
a3-2         PARAGERAHAPATPRRARPPPAAAAAAAPIX   536
             *.*       *       ***
```

Supplemental Figure S4. Deduced amino acid sequences of *CYP735A3*, *cyp735a3-1* and *cyp735a3-2*
Deduced amino acid sequences of *CYP735A3*, *cyp735a3-1* (*a3-1*), and *cyp735a3-2* (*a3-2*) were aligned by Clustal Omega (https://www.ebi.ac.uk/Tools/msa/clustalo/). Triangles indicate CRISPR target sites.

```
                                  g4-1-1        g4-2-1
                                    v             v
CYP735A4        MAVLVSLMVIAASSPLVALLLRAAWVTLSCYWLTPMRIRRAMAAQGVRGPPPRPLVGNLR      60
a4-1            MAVLVSLMVIAASSPLVALLLIVRSF-VDS------------------------------      17
a4-2            MAVLVSLMVIAASSPLVALLLRAAWVTLSCYWLTPVPD----------------------      38
                *****************

                                            g4-1-2             g4-2-2
                                              v                  v
CYP735A4        EVSALVARATADDMPSLSHDIVGRLMPHYVLWSGTYGKLFVYLYGSEPRLCLTDTALIKE      120
a4-1            ------------------------------FW--FAGKLFVYLYGSEPRLCLTDTALIKE      45
a4-2            ------------------------------------------------------------      38

CYP735A4        FLSSKYAHATGKSWLQRQGTKHFIGGGLLMANGARWAHQRHVVAPAFMADKLKARGRVGR      180
a4-1            FLSSKYAHATGKSWLQRQGTKHFIGGGLLMANGARWAHQRHVVAPAFMADKLKARGRVGR      105
a4-2            ------------------------------------------------------------      38

CYP735A4        MVECTKQAIRELRDAAAGRRGEEVEIGAHMTRLTGDIISRTEFNTSYDTGKRIFLLLEHL      240
a4-1            MVECTKQAIRELRDAAAGRRGEEVEIGAHMTRLTGDIISRTEFNTSYDTGKRIFLLLEHL      165
a4-2            ------------------------------------------------------------      38

CYP735A4        QRLTSRSSRHLWIPGSQYFPSKYRREIRRLNGELEAVLMESIRRSREIADEGRAAVATYG      300
a4-1            QRLTSRSSRHLWIPGSQYFPSKYRREIRRLNGELEAVLMESIRRSREIADEGRAAVATYG      225
a4-2            ------------------------------------------------------------      38

CYP735A4        RGLLAMLLSEMEEKEKNGGGGGGEFSYDAQLVIDECKTFFFAGHETSALLLTWAIMLLAT      360
a4-1            RGLLAMLLSEMEEKEKNGGGGGGEFSYDAQLVIDECKTFFFAGHETSALLLTWAIMLLAT      285
a4-2            ------------------------------------------------------------      38

CYP735A4        NPAWQEKARTEVAAVCGDHPPSADHLSKLTVLQMIIQETLRLYPPATLLPRMAFEDIQLG      420
a4-1            NPAWQEKARTEVAAVCGDHPPSADHLSKLTVLQMIIQETLRLYPPATLLPRMAFEDIQLG      345
a4-2            ------------------------------------------------------------      38

CYP735A4        GLRLPRGLSVWIPVLAIHHDESIWGPDAHEFRPERFAPGARRPSAAGAARFLPFAAGPRN      480
a4-1            GLRLPRGLSVWIPVLAIHHDESIWGPDAHEFRPERFAPGARRPSAAGAARFLPFAAGPRN      405
a4-2            ------------------------------------------------------------      38

CYP735A4        CVGQAYALVEAKVVLAMLLSAFRFAISDNYRHAPENVLTLRPKHGVPVHLRPLRP      535
a4-1            CVGQAYALVEAKVVLAMLLSAFRFAISDNYRHAPENVLTLRPKHGVPVHLRPLRP      460
a4-2            -------------------------------------------------------      38
```

Supplemental Figure S5. Deduced amino acid sequences of *CYP735A4*, *cyp735a4-1* and *cyp735a4-2*
Deduced amino acid sequences of *CYP735A4*, *cyp735a4-1* (*a4-1*), and *cyp735a4-2* (*a4-2*) were aligned by Clustal Omega (https://www.ebi.ac.uk/Tools/msa/clustalo/). Triangles indicate CRISPR target sites. The “A helix is highlighted in blue.

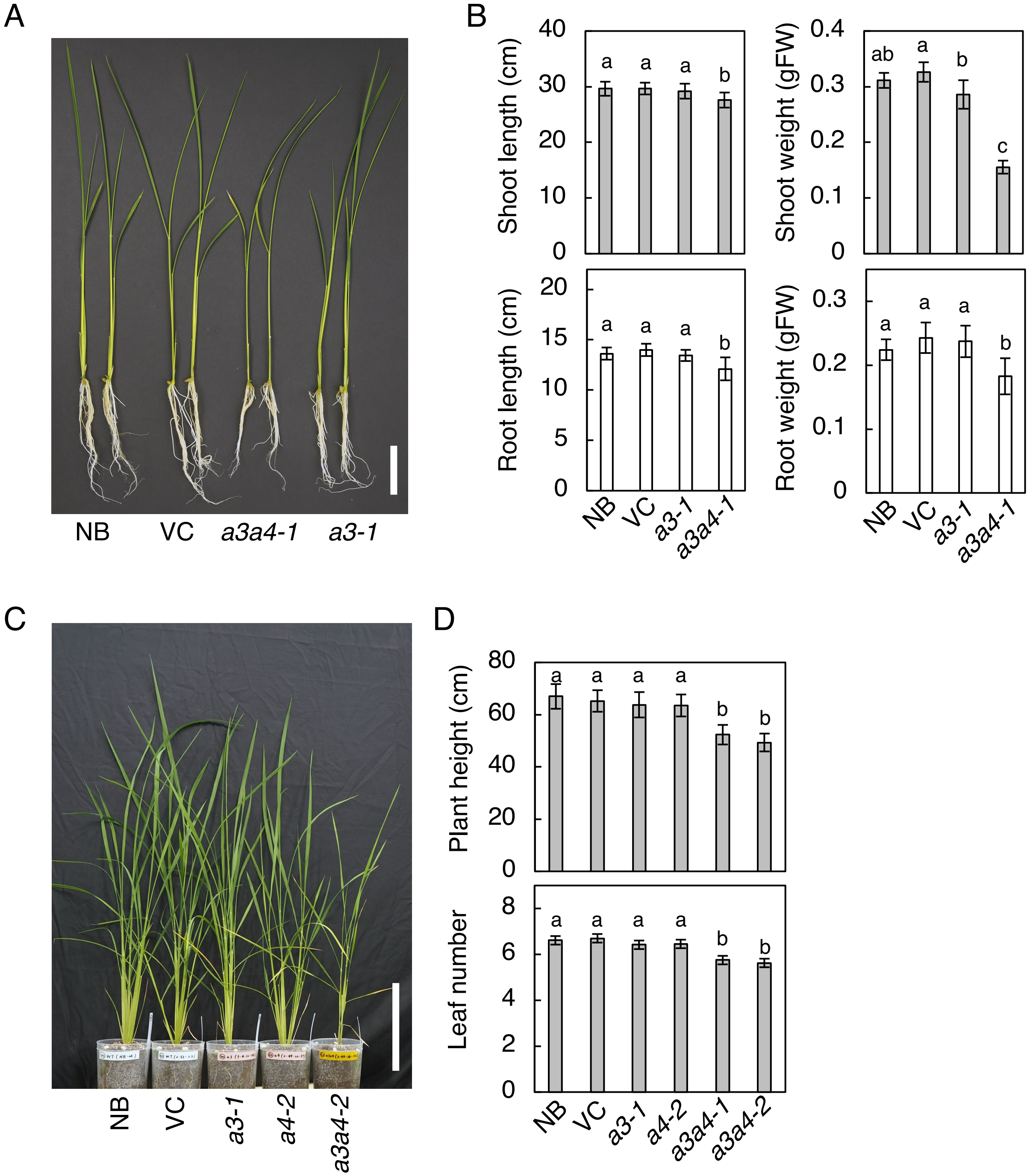

Supplemental Figure S6. Effect of disruption of *CYP735A*s on vegetative growth
(A) A representative image of Nipponbare (NB), vector control (VC), *cyp735a3-1 cyp735a4-1* (*a3a4-1*), and *cyp735a3-1* (*a3-1*) seedlings grown 14 days in hydroponic culture. Scale bar, 5 cm. (B) Quantification of shoot length, shoot fresh weight, root length, and root fresh weight of 14 day-old seedlings. NB, VC, *cyp735a3-1* (*a3-1*), and *a3a4-1* seedlings were grown in hydroponic culture. (C) A representative image of NB, VC, *a3-1*, *cyp735a4-1* (*a4-2*), *cyp735a3-2 cyp735a4-2* (*a3a4-2*) seedlings grown for 43 days on soil. Scale bar, 20 cm. (D) Plant height and leaf number of NB, VC, *a3-1*, *a4-2*, *a3a4-1*, and *cyp735a3-2 cyp735a4-2* (*a3a4-2*) grown for 30 days on soil. Error bars represent standard deviation of biological replicates (B, n=6-13; D, n=9-12). Different lowercase letters indicate statistically significant differences as indicated by Tukey's HSD test (p<0.05).

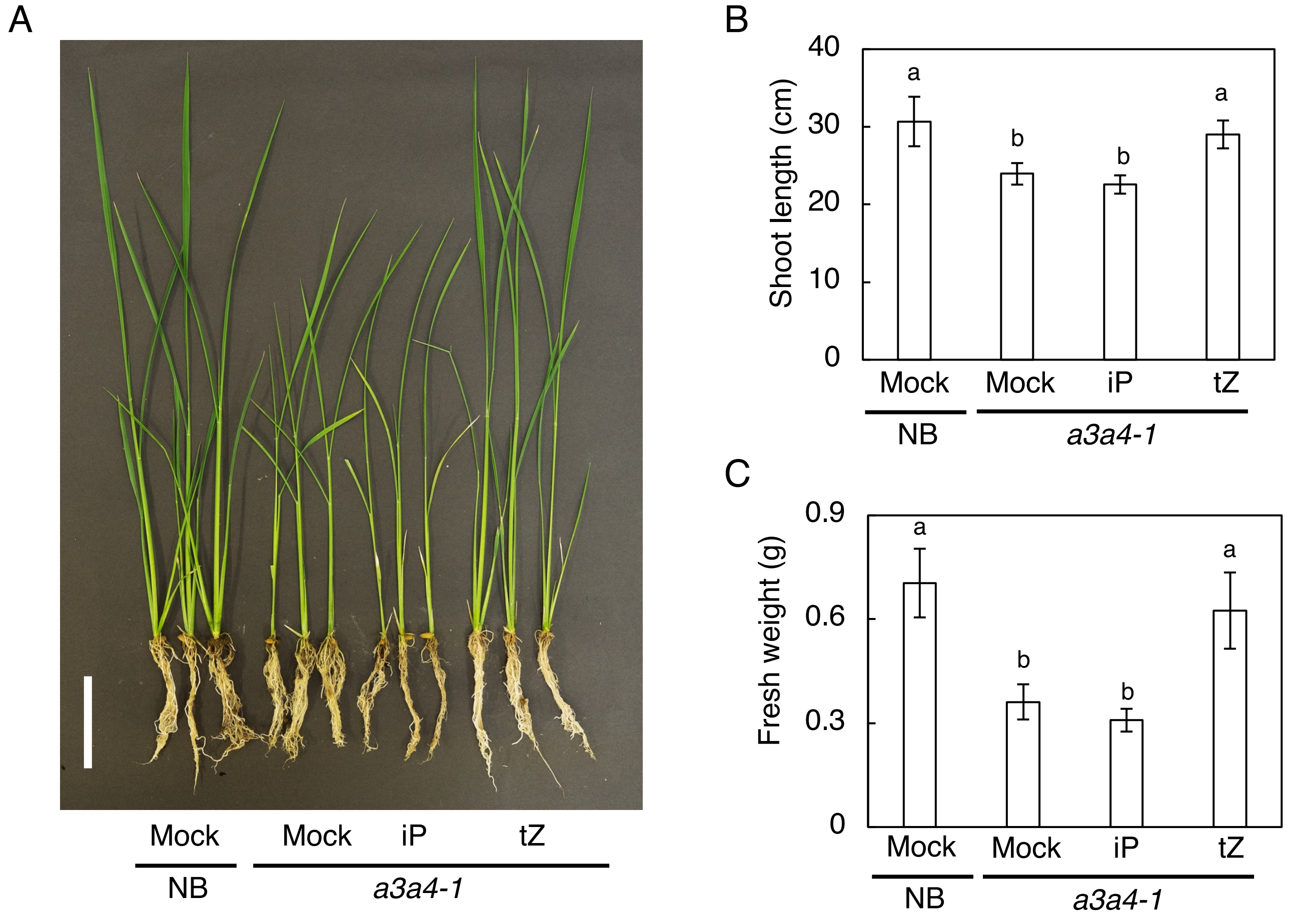

Supplemental Figure S7. External application of tZ rescues the growth defect of *cyp735a3 cyp735a4* seedlings

(A) A representative image of 14-day-old Nipponbare (NB) and *cyp735a3-1 cyp735a4-1* (*a3a4-1*) sprayed with 0.01% (v/v) DMSO (Mock), 20 μM iP (iP) or 20 μM tZ (tZ) solutions daily. Scale bar, 5 cm. (B, C) Quantification of shoot length (B) and fresh weight (C) of the 14-day-old NB and *a3a4-1* sprayed with Mock, iP or tZ solutions daily. Error bars represent standard deviation of biological replicates (n>7). Different lowercase letters indicate statistically significant differences as indicated by Tukey's HSD test (p<0.05).

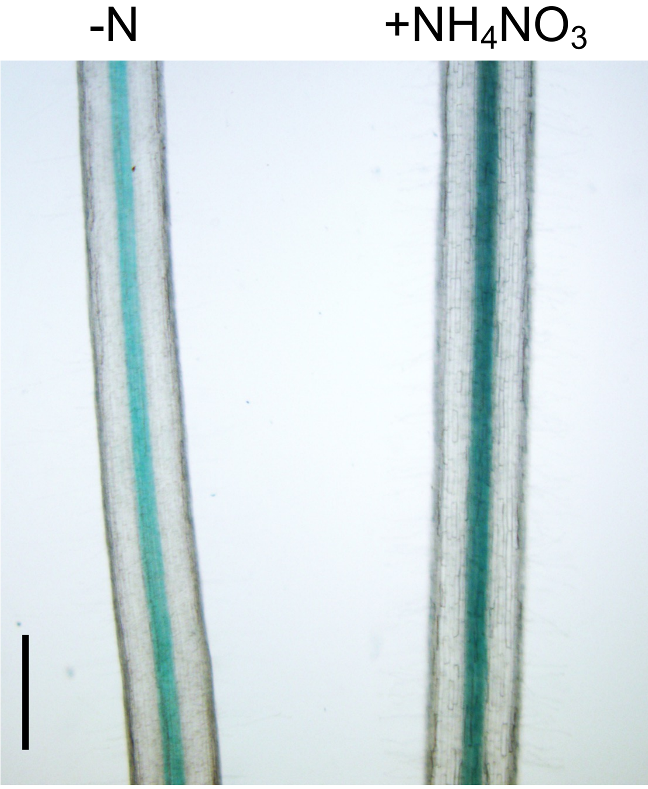

Supplemental Figure S8. A representative image of GUS staining obtained from a proCYP735A3:GUS transgenic seedling before and after ammonium nitrate supplementation

Sixteen-day-old proCYP735A3:GUS transgenic seedlings hydroponically grown in nitrogen-free nutrient solution were incubated with the nutrient solution supplemented with 5 mM ammonium nitrate for 24 h. Crown roots were harvested from each seedling before (-N) and 24 h after (+NH_4NO_3) ammonium nitrate supplementation and were subjected to GUS staining. Scale bar, 200 μm.

Table S1. Cytokinin concentrations in Col-0, *cyp735a1 cyp735a2* mutant, and transgenic plants constitutively expressing *CYP735A3* (*A3*-ox) or *CYP735A4* (*A4*-ox) in *cyp735a1 cyp735a2* background.

pmol/gFW	Col-0	*cyp735a1 cyp735a2*	*A3*-ox (Line 2)	*A4*-ox (Line 2)
tZ	0.60 ± 0.10*	0.05 ± 0.01	32.83 ± 13.72*	48.64 ± 4.57*
tZR	1.62 ± 0.23*	0.07 ± 0.02	15.37 ± 3.73*	21.59 ± 2.86*
tZRPs	20.02 ± 2.11*	1.01 ± 0.10	17.42 ± 3.20*	19.77 ± 1.51*
cZ	0.12 ± 0.01	0.2 ± 0.07	0.62 ± 0.49	0.78 ± 0.27
cZR	0.57 ± 0.15	0.61 ± 0.15	2.54 ± 2.50	4.10 ± 1.51
cZRPs	4.19 ± 0.5	4.72 ± 1.23	2.71 ± 1.33	2.42 ± 1.13
iP	N.D.	0.12 ± 0.00	N.D.	N.D.
iPR	0.66 ± 0.09	0.98 ± 0.26	N.D.	N.D.
iPRPs	33.48 ± 5.59*	68.77 ± 12.63	2.97 ± 0.99*	2.08 ± 0.11*
tZ7G	18.95 ± 0.37*	1.56 ± 0.11	31.5 ± 14.94*	19.46 ± 1.13*
tZ9G	8.93 ± 0.83*	0.33 ± 0.07	16.81 ± 9.75*	8.40 ± 0.57*
tZOG	12.82 ± 1.22*	0.75 ± 0.13	17.30 ± 8.24*	9.54 ± 0.95*
cZOG	2.8 ± 0.5*	5.03 ± 1.27	0.95 ± 0.75*	0.56 ± 0.30*
tZROG	0.79 ± 0.12*	0.05 ± 0.02	1.55 ± 0.87*	0.94 ± 0.05*
cZROG	1.09 ± 0.05	1.18 ± 0.20	0.75 ± 0.39	0.6 ± 0.32
tZRPsOG	0.38 ± 0.08	N.D.	0.53 ± 0.27	0.31 ± 0.08
cZRPsOG	N.D.	N.D.	N.D.	N.D.
iP7G	15.17 ± 0.6*	35.66 ± 1.40	1.13 ± 0.51*	0.73 ± 0.03*
iP9G	2.78 ± 0.10*	7.39 ± 0.22	0.15 ± 0.11*	0.07 ± 0.01*
iP-type	52.08 ± 5.89*	112.92 ± 12.58	4.25 ± 1.61*	2.89 ± 0.12*
tZ-type	64.1 ± 3.41*	3.81 ± 0.32	133.3 ± 14.42*	128.67 ± 9.51*
cZ-type	8.76 ± 0.90	11.73 ± 2.71	7.42 ± 2.71	8.47 ± 2.41
Total CK	124.94 ± 7.37	128.45 ± 12.5	144.97 ± 13.69	140.03 ± 7.17

Seedlings were grown for 12 days on 1/2x MS agart plates and whole seedlings were harvested. Data are means ± standard deviation (n = 4). gFW, gram fresh weight; *cyp735a1 cyp735a2, cyp735a1-2 cyp735a2-2*; tZ, *trans*-zeatin; tZR ,tZ riboside; tZRPs, tZ ribotides; cZ, *cis*-zeatin; cZR, cZ riboside; cZRPs, cZ ribotides; iP, *N*6-(Δ2-isopentenyl)adenine; iPR, iP riboside; iPRPs, iP ribotides; tZ7G, tZ-7-*N*-glucoside; tZ9G, tZ-9-*N*-glucoside; tZOG, tZ-*O*-glucoside; cZOG, cZ-*O*-glucoside; tZROG, tZR-*O*-glucoside; cZROG, cZR-*O*-glucoside; iP7G, iP-7-*N*-glucoside; iP9G, iP-9-*N*-glucoside, N.D., under the quantification detection limit.. *, significantly different from *cyp735a1 cyp735a2* in Student's *t*-test (p<0.05)

Table S2. Cytokinin concentrations in Nipponbare, vector control, *cyp735a3-1* single mutant, and *cyp735a3-1 cyp735a4-1* double mutant.

	Shoot				Root			
pmol/gFW	Nipponbare	Vector control	*cyp735a3-1*	*cyp735a3-1 cyp735a4-1*	Nipponbare	Vector control	*cyp735a3-1*	*cyp735a3-1 cyp735a4-1*
tZ	0.34 ± 0.15	0.34 ± 0.16	0.15 ± 0.17	N.D.	0.04 ± 0.05	0.14 ± 0.05	0.08 ± 0.06	N.D.
tZR	0.12 ± 0.07	0.09 ± 0.06	0.09 ± 0.07	0.01 ± 0.02*	0.07 ± 0.02	0.04 ± 0.01	0.04 ± 0.01	0.01 ± 0.01*
tZRPs	0.1 ± 0.08	0.12 ± 0.04	0.08 ± 0.04	N.D.	0.35 ± 0.14	0.33 ± 0.07	0.27 ± 0.11	0.03 ± 0.03*
cZ	1.28 ± 0.14	2.43 ± 1.45	1.62 ± 0.49	2.15 ± 0.84	2.15 ± 0.67	1.67 ± 0.64	2.19 ± 0.88	2.04 ± 0.23
cZR	1.53 ± 0.28	2.81 ± 1.81	1.69 ± 0.35	1.87 ± 0.34	1.99 ± 0.56	1.62 ± 0.42	1.99 ± 0.66	2.08 ± 0.55
cZRPs	0.62 ± 0.14	0.86 ± 0.10	0.74 ± 0.16	1.12 ± 0.43	2.59 ± 0.59	2.53 ± 0.62	3.36 ± 1.34	2.85 ± 0.97
iP	0.40 ± 0.06	0.36 ± 0.06	0.39 ± 0.11	0.69 ± 0.27	0.28 ± 0.07	0.23 ± 0.05	0.2 ± 0.02	0.28 ± 0.05
iPR	0.06 ± 0.00	0.24 ± 0.25	0.08 ± 0.03	0.11 ± 0.04	0.30 ± 0.09	0.28 ± 0.04	0.42 ± 0.08	0.93 ± 0.31*
iPRPs	0.56 ± 0.08	0.46 ± 0.1	0.49 ± 0.12	1.13 ± 0.31*	1.47 ± 0.25	1.33 ± 0.2	2.02 ± 0.64	2.37 ± 0.33*
DZ	N.D.	N.D.	N.D.	N.D.	N.D.	N.D.	N.D.	N.D.
DZR	0.05 ± 0.01	0.06 ± 0.02	0.05 ± 0.02	0.04 ± 0.02	N.D.	N.D.	N.D.	N.D.
DZRPs	N.D.	N.D.	N.D.	N.D.	N.D.	N.D.	N.D.	N.D.
tZ7G	N.D.	N.D.	N.D.	N.D.	N.D.	0.01 ± 0.02	N.D.	N.D.
tZ9G	7.11 ± 1.89	9.19 ± 2.69	5.57 ± 2.48	0.07 ± 0.02*	5.07 ± 1.26	7.18 ± 2.26	4.29 ± 0.87	0.05 ± 0.01*
tZOG	0.18 ± 0.04	0.13 ± 0.10	0.24 ± 0.03	0.32 ± 0.03*	0.14 ± 0.03	0.13 ± 0.03	0.13 ± 0.05	0.13 ± 0.07
cZOG	318.42 ± 20.50	310.04 ± 101.95	326.98 ± 33.68	291.78 ± 42.49	154.34 ± 5.81	147.97 ± 19.26	165.49 ± 29.53	126.67 ± 24.20
tZROG	0.1 ± 0.02	0.09 ± 0.01	0.08 ± 0.03	0.11 ± 0.03	0.29 ± 0.07	0.27 ± 0.05	0.23 ± 0.04	0.19 ± 0.03
cZROG	40.34 ± 0.86	36.09 ± 2.53	38.38 ± 1.98	37.19 ± 2.74	64.04 ± 3.96	60.04 ± 6.87	66.87 ± 11.01	72.92 ± 7.60
tZRPsOG	N.D.	N.D.	N.D.	N.D.	N.D.	N.D.	N.D.	N.D.
cZRPsOG	2.71 ± 0.25	2.87 ± 0.25	2.52 ± 0.10	3.16 ± 0.36	2.37 ± 0.58	2.58 ± 0.75	2.94 ± 0.33	2.87 ± 0.46
iP7G	N.D.	N.D.	N.D.	N.D.	N.D.	0.05 ± 0.08	N.D.	N.D.
iP9G	0.94 ± 0.13	1.17 ± 0.13	1.25 ± 0.31	2.22 ± 0.58*	1.69 ± 0.18	1.91 ± 0.46	1.76 ± 0.26	3.76 ± 1.01*
DZ9G	0.25 ± 0.03	0.19 ± 0.01	0.18 ± 0.01	0.14 ± 0.02*	0.08 ± 0.01	0.08 ± 0.01	0.07 ± 0.01	0.04 ± 0.00*
tZ-type	7.96 ± 2.07	9.97 ± 3.00	6.20 ± 2.59	0.51 ± 0.07*	5.96 ± 1.35	8.10 ± 2.36	5.04 ± 0.93	0.41 ± 0.11*
iP-type	1.95 ± 0.19	2.22 ± 0.40	2.22 ± 0.52	4.14 ± 1.00*	3.74 ± 0.52	3.80 ± 0.63	4.41 ± 0.87	7.34 ± 1.51*
cZ-type	364.88 ± 19.89	355.1 ± 108.00	371.93 ± 34.80	337.27 ± 41.77	227.48 ± 9.92	216.39 ± 24.81	242.86 ± 40.34	209.42 ± 28.31
DZ-type	0.30 ± 0.02	0.25 ± 0.03	0.24 ± 0.02*	0.18 ± 0.04*	0.08 ± 0.01	0.08 ± 0.01	0.07 ± 0.01	0.04 ± 0.00*
iP-type + tZ-type	9.91 ± 2.24	12.19 ± 3.30	8.42 ± 2.94	4.65 ± 1.04*	9.7 ± 1.85	11.91 ± 2.63	9.45 ± 1.51	7.76 ± 1.55
Total CK	375.09 ± 18.05	367.54 ± 110.6	380.58 ± 36.78	342.1 ± 42.58	237.27 ± 11.31	228.37 ± 24.84	252.37 ± 41.75	217.22 ± 28.65

Seedlings were grown hydroponically for 15 days and shoots and roots were harvested separately. Data are means ± standard deviation (n = 3-6). gFW, gram fresh weight; , tZ, *trans*-zeatin; tZR ,tZ riboside; tZRPs, tZ ribotides; cZ, cis-zeatin; cZR, cZ riboside; cZRPs, cZ ribotides; iP, *N*6-(Δ2-isopentenyl)adenine; iPR, iP riboside; iPRPs, iP ribotides; DZ, dihydrozeation; DZR, dihydrozeation riboside; DZRPs. dihydrozeation ribotide; tZ7G, tZ-7-*N*-glucoside; tZ9G, tZ-9-*N*-glucoside; tZOG, tZ-*O*-glucoside; cZOG, cZ-*O*-glucoside; tZROG, tZR-*O*-glucoside; cZROG, cZR-*O*-glucoside; DZ9G, DZ-9-*N*-glucoside; iP7G, iP-7-*N*-glucoside; iP9G, iP-9-*N*-glucoside, N.D., under the quantification detection limit. *, significantly different from Nipponbare in Student's *t*-test ($p<0.05$)

Table S3. Cytokinin concentrations in Nipponbare, vector control, *cyp735a4-2* single mutant, and *cyp735a3-2 cyp735a4-2* double mutant.

	Shoot				Root			
pmol/gFW	Nipponbare	Vector control	*cyp735a4-2*	*cyp735a3-2 cyp735a4-2*	Nipponbare	Vector control	*cyp735a4-2*	*cyp735a3-2 cyp735a4-2*
tZ	0.37 ± 0.05	0.48 ± 0.11	0.34 ± 0.13	N.D.	0.12 ± 0.02	0.07 ± 0.06	0.04 ± 0.04**	N.D.
tZR	0.18 ± 0.06	0.29 ± 0.09	0.15 ± 0.09	0.01 ± 0.02*	0.05 ± 0.03	0.05 ± 0.05	0.02 ± 0.02	N.D.
tZRPs	0.88 ± 0.23	0.86 ± 0.18	0.85 ± 0.18	0.03 ± 0.04*	0.38 ± 0.13	0.38 ± 0.23	0.20 ± 0.10	0.08 ± 0.06
cZ	5.67 ± 10.21	1.35 ± 0.31	1.55 ± 0.2	1.31 ± 0.28	0.45 ± 0.19	0.30 ± 0.06	0.56 ± 0.51	0.34 ± 0.15
cZR	3.2 ± 2.03	2.13 ± 0.21	2.41 ± 0.45	2.41 ± 0.23	1.61 ± 0.40	1.55 ± 0.15	1.99 ± 0.47	1.88 ± 0.19*
cZRPs	1.22 ± 0.41	1.16 ± 0.22	1.22 ± 0.15	1.56 ± 0.18	3.62 ± 2.65	2.22 ± 0.42	3.41 ± 2.05	3.34 ± 0.92
iP	0.71 ± 0.4	1.27 ± 0.73	1.77 ± 0.74*	1.25 ± 0.32*	N.D.	N.D.	N.D.	0.04 ± 0.1
iPR	0.29 ± 0.13	0.57 ± 0.31	0.45 ± 0.14*	0.66 ± 0.21*	0.32 ± 0.08	0.17 ± 0.06*	0.57 ± 0.22	0.98 ± 0.29*
iPRPs	1.34 ± 0.33	1.75 ± 0.7	2.7 ± 0.71*	3.65 ± 0.61*	0.67 ± 0.13	0.61 ± 0.2	1.44 ± 0.43*	2.02 ± 0.47*
DZ	N.D.	N.D.	N.D.	N.D.	N.D.	N.D.	N.D.	N.D.
DZR	0.10 ± 0.03	0.07 ± 0.01	0.06 ± 0.02*	0.05 ± 0.01*	N.D.	N.D.	N.D.	N.D.
DZRPs	N.D.	N.D.	N.D.	N.D.	N.D.	N.D.	N.D.	N.D.
tZ7G	N.D.	N.D.	N.D.	N.D.	N.D.	N.D.	N.D.	N.D.
tZ9G	11.22 ± 1.23	9.68 ± 0.81*	5.85 ± 0.95*	0.11 ± 0.08*	4.50 ± 0.67	4.11 ± 1.06	2.26 ± 0.96*	0.08 ± 0.03*
tZOG	0.17 ± 0.08	0.17 ± 0.07	0.22 ± 0.06	0.21 ± 0.2	0.03 ± 0.05	0.21 ± 0.06*	0.14 ± 0.13	0.09 ± 0.08*
cZOG	223.14 ± 52.14	228.01 ± 45.08	250.02 ± 46.71	270.67 ± 34.6	184.07 ± 12.17	166.69 ± 11.65*	197.41 ± 33.84	144.06 ± 59.64
tZROG	0.03 ± 0.05	0.02 ± 0.04	0.07 ± 0.07	0.09 ± 0.06	0.25 ± 0.12	0.32 ± 0.04	0.24 ± 0.06	0.22 ± 0.04*
cZROG	26.66 ± 2.94	26.46 ± 2.72	28.32 ± 3.84	31.67 ± 2.88*	52.24 ± 3.32	54.92 ± 3.81	56.25 ± 4.71	58.68 ± 4.39
tZRPsOG	N.D.	N.D.	N.D.	N.D.	N.D.	N.D.	N.D.	N.D.
cZRPsOG	2.38 ± 0.23	3.06 ± 0.59*	3.34 ± 0.45*	3.77 ± 0.77*	2.48 ± 0.33	3.82 ± 0.91	4.25 ± 1.24*	4.94 ± 0.92*
iP7G	N.D.	N.D.	N.D.	N.D.	N.D.	N.D.	N.D.	N.D.
iP9G	3.78 ± 0.51	4.09 ± 0.95	5.12 ± 0.8*	6.21 ± 1.07*	1.48 ± 0.24	1.23 ± 0.13	2.19 ± 0.58	4.44 ± 1.18*
DZ9G	0.17 ± 0.02	0.17 ± 0.02	0.19 ± 0.03	0.20 ± 0.03	0.09 ± 0.02	0.08 ± 0.01	0.08 ± 0.01	0.08 ± 0.01
tZ-type	12.86 ± 1.48	11.5 ± 0.70	7.48 ± 1.04*	0.45 ± 0.26*	5.33 ± 0.90	5.15 ± 0.94	2.89 ± 1.09*	0.47 ± 0.13*
iP-type	6.11 ± 1.23	7.69 ± 2.29	10.04 ± 2.12*	11.77 ± 1.12*	2.48 ± 0.42	2.00 ± 0.28	4.20 ± 1.19*	7.48 ± 1.72*
cZ-type	262.26 ± 52.20	262.17 ± 47.75	286.87 ± 45.62	311.38 ± 32.49	244.48 ± 12.02	229.49 ± 12.73	263.87 ± 38.36	213.25 ± 57.99
DZ-type	0.22 ± 0.11	0.24 ± 0.02	0.24 ± 0.03	0.24 ± 0.04	0.09 ± 0.02	0.08 ± 0.01	0.08 ± 0.01	0.08 ± 0.01
iP-type + tZ-type	18.98 ± 1.85	19.18 ± 1.29	17.52 ± 2.00	12.22 ± 1.30*	7.81 ± 1.29	7.15 ± 1.07	7.09 ± 2.21	7.95 ± 1.82
Total	281.46 ± 53.57	281.59 ± 48.84	304.63 ± 46.94	323.83 ± 31.67	252.38 ± 12.42	236.71 ± 11.99	271.05 ± 39.98	221.28 ± 56.68

Seedlings were grown hydroponically for 13 days and shoots and roots were harvested separately. Data are means ± standard deviation (n = 3-6). gFW, gram fresh weight; , tZ, *trans*-zeatin; tZR ,tZ riboside; tZRPs, tZ ribotides; cZ, *cis*-zeatin; cZR, cZ riboside; cZRPs, cZ ribotides; iP, *N*6-(Δ2-isopentenyl)adenine; iPR, iP riboside; iPRPs, iP ribotides; DZ, dihydrozeation; DZR, dihydrozeation riboside; DZRPs. dihydrozeation ribotide; tZ7G, tZ-7-*N*-glucoside; tZ9G, tZ-9-*N*-glucoside; tZOG, tZ-*O*-glucoside; cZOG, cZ-*O*-glucoside; tZROG, tZR-*O*-glucoside; cZROG, cZR-*O*-glucoside; DZ9G, DZ-9-*N*-glucoside; iP7G, iP-7-*N*-glucoside; iP9G, iP-9-*N*-glucoside, N.D., under the quantification detection limit. *, significantly different from Nipponbare in Student's *t*-test ($p<0.05$)

Table S4. List of primers used for vector construction and genotyping.

Name (Forward/Reverse)	Purpose	Forward (5' to 3')	Reverse (5' to 3')
oxCYP735A3-F/R	Construction of pBI121-CYP735A3	TTTCTAGATGGCAATGGCCGCCGCCGT	CTATGGGCGCGGCGGCGGCAGCGG
oxCYP735A4-F/R	Construction of pBI121-CYP735A4	TTTCTAGATGGCGGTCCTCGTGTCGCTCA	CTATGGCCGCAGCGGCCGGAGG
gCYP735A3A4-1-1-F/R	Construction of pMgPoef4_129-2A-GFP-cyp735a3a4-1	TTGGGTCTCGTGCACTCATGCTACTACCTCACGCGTTTTAGAGCTAGAAATAGCA	TTGGGTCTCCAGCAGCGCCACGTGCACCAGCCGGGAATCGAA
gCYP735A3A4-1-2-F/R	Construction of pMgPoef4_129-2A-GFP-cyp735a3a4-1	TTGGGTCTCGTGCTTCTGAGGGGTTTTAGAGCTAGAAATAGCA	TTGGGTCTCCAAACGCGATTGTTCGTTCGTTCGTTGCACCAGCCGGGAATCGAA
gCYP735A3A4-2-1-F/R	Construction of pMgPoef4_129-2A-GFP-cyp735a3a4-2	TTGGGTCTCGTGCACGGCGGGTCCTGGCGAGCCAGTTTTAGAGCTAGAAATAGCA	TTGGGTCTCCTCAGCCAGTAGCTGCACCAGCCGGGAATCGAA
gCYP735A3A4-2-2-F/R	Construction of pMgPoef4_129-2A-GFP-cyp735a3a4-2	TTGGGTCTCGCTGACGCCAATGGTTTTAGAGCTAGAAATAGCA	TTGGGTCTCCAAACAGGCTGTGCCTGACTGACACTGCACCAGCCGGGAATCGAA
gCYP735A3-F2/R2	Genotyping of *cyp735a3* mutations	TCCTCGTCGCCATCGCATTG	TTGCTCGTGAATGGGAGGAGC
gCYP735A4-F3/R3	Genotyping of *cyp735a4* mutations	ACATGCCGTCCCTCAGCCAC	GGCGTGCTCACCGTATGTCC
proCYP735A3-F/R	Construction of pCAMBIA1390-proCYP735A3:GUS	CACCACGTCGAACACCTGGTGGCCG	ATGGCCGCCGCCGTCCTCGTC
proCYP735A4-F/R	Construction of pCAMBIA1390-proCYP735A4:GUS	CACCGCGCACCACCACATGCACACACG	GAGCGACACGAGGACCGCCAT

Table S5. List of primers used for quantitative RT-PCR analysis.

Gene name	Locus ID	Forward (5' to 3')	Reverse (5' to 3')
ACT8	AT1G49240	AACATTGTGCTCAGTGGTGG	GTGGTGCCACGACCTTAATC
CYP735A3	LOC_Os08g33300/Os08g0429800	TACGAGACCGGCAAGAGGAT	GCTCGGAAAATACTGGCTGC
CYP735A4	LOC_Os09g23820/Os09g0403300	CGCGCTGATCAAGGAGTTCT	CAGTGTCGTAGCTGGTGTTG
ZIURP1	LOC_Os03g08010/Os03g0234200	CACCCTAGGGCTGTCAACTG	GCGAGTGACGCTCTAGTTCT
OsRR1	LOC_Os04g36070/Os04g0442300	GTCTCTCGCCTTGTCACCTT	AGAAGCGAAGCACTGATCCT
OsRR2	LOC_Os02g35180/Os02g0557800	GTCGCACTACTTCCAGCTCA	GAATTCATGCGCACCACAGG
OsRR4	LOC_Os01g72330/Os01g0952500	TGAGAATGTGCCTGCAAGGAT	CAGCGAGCTTGACAGGTTTC
OsRR6	LOC_Os04g57720/Os04g0673300	GTGGTGATCATGTCGTCGGA	ATCTGATACGGCTGCAGAGC
OsRR9	LOC_Os11g04720/Os11g0143300	CTCTGGAGTTCTTGGGGCTC*	CCAGGCATGCAGTAGTCTGT*
OsRR10	LOC_Os12g04500/Os12g0139400		
OsRR21	LOC_Os03g12350/Os03g0224200	GAGTCACAGCATTGGAAGCAG	TGTTTCCAGTGATTACCCTACTTA
OsRR23	LOC_Os02g55320/Os02g0796500	CAGACTACAGAGGGATGGCG	AAATGGTGGAGGGCAACAGA
OsSIPP2C1	LOC_Os09g15670/Os09g0325700	GTCACCCAGCTGATGCTGTA	CACTGAACTTGGTTAATTCAGGGA
OsGH3.2	LOC_Os01g55940/Os01g0764800	GCAAGGGGCTCTACTTCCTG	GTAGTAGCTGGTCAGCACCG
OsIPT4	LOC_Os03g59570/Os03g0810100	GTAGATCTCGAGGTGCTCCG	AGATGCCCCTGGAGTAGTCG
OsNIA1	LOC_Os08g36480/Os08g0468100	CGTGGACCGTCGATGTGAC	CATGTTCTGCTCCTTGCGG
